# Identification of divergent isolates of cherry latent virus 1 in Greek sweet cherry orchards

**DOI:** 10.1007/s00705-023-05875-7

**Published:** 2023-09-07

**Authors:** Chrysoula G. Orfanidou, Asimina Katsiani, Thierry Candresse, Armelle Marais, Theodora Gkremotsi, Pavlina Drogoudi, Konstantinos Kazantzis, Nikolaos I. Katis, Varvara I. Maliogka

**Affiliations:** 1 Laboratory of Plant Pathology, Faculty of Agriculture, Forestry and Natural Environment, School of Agriculture, Aristotle University of Thessaloniki, Thessaloniki, 54124 Greece; 2 Univ. Bordeaux, INRAE, UMR 1332 Biologie du Fruit et Pathologie, Villenave d’Ornon, France; 3 Department of Deciduous Fruit trees, Institute of Plant Breeding and Genetic Resources, Hellenic Agricultural Organization – ‘DIMITRA’, 38 R.R. Station, Naoussa, 59035 Greece

**Keywords:** trichovirus, cherry latent virus 1, high-throughput sequencing, genetic diversity, virome

## Abstract

In this study, samples collected from eight sweet cherry trees in northern Greece were analyzed by high-throughput sequencing for the presence of viruses. Bioinformatic analysis revealed the presence of divergent isolates of cherry latent virus 1 (CLV-1), a recently identified trichovirus in a sweet cherry accession imported into the USA from the Republic of Georgia. The complete genome sequences of seven CLV-1 isolates were determined, and phylogenetic analysis indicated that they belonged to a separate clade from the previously characterized Georgian isolate. A small-scale survey confirmed the presence of CLV-1 in 47 out of 151 sweet cherry samples tested, and partial sequencing of 15 isolates showed a high degree of nucleotide sequence similarity among them.

**Supplementary Information:**

The online version contains supplementary material available at 10.1007/s00705-023-05875-7.

Sweet cherry (*Prunus avium* L.) is an economically important fruit crop worldwide. In 2019, the total cherry production in Greece was 81,600 tons from approximately 16,100 hectares, thus ranking the country 7th in the world in cherry production, after Turkey, the USA, Chile, Uzbekistan, Iran, and Italy [1]. A number of viruses have been reported to infect sweet cherry, with the majority of them causing latent infections. Viruses such as prune dwarf virus (PDV, genus *Ilarvirus*) and prunus necrotic ringspot virus (PNRSV, genus *Ilarvirus*) can affect cherry production by causing crop losses from 18 to 30% [2], whereas other viruses, such as cherry leaf roll virus (CLRV, genus *Nepovirus*), can cause severe losses that reach 91–98% [3]. Two trichoviruses, apple chlorotic leaf spot virus (ACLSV) and cherry mottle leaf virus (CMLV) [4, 5], have also been detected in cherry trees. CMLV and ACLSV infections are usually latent, but on highly susceptible sweet cherry cultivars and depending on the viral isolate, severe symptoms can be observed [6, 7].

In 2020, a novel trichovirus was detected with the aid of high-throughput sequencing (HTS) technology in a symptomless sweet cherry accession imported into the USA from the Republic of Georgia [8] and was provisionally named cherry latent virus 1 (CLV-1). The complete genome sequence of CLV-1 consists of 7,460 nucleotides, excluding the 3′ poly(A) tail. Its genome organization is very similar to that of several trichoviruses infecting fruit trees, with three open reading frames encoding a putative RNA-dependent-RNA-polymerase (RdRp), a movement protein (MP), and a coat protein (CP) [8]. In the USA, 175 cherry germplasm samples from flowering, sour, and sweet cherries were tested for the presence of CLV-1, but the virus was not detected, indicating that it is rather uncommon in cherry genetic resources in that country [8].

In this study, samples of plant material collected from eight sweet cherry trees grown in orchards in Greece (Supplementary Table S1) were analyzed individually by HTS for the presence of known and unknown viruses. Among these samples, five were collected within the framework of this study (2019–2020), and the other three were collected during past surveys (2009, 2014). Total RNA extraction, library preparation, Illumina sequencing, and bioinformatic analysis were performed as described previously [9] using dried leaf tissue. In order to confirm the correctness of the *de novo* assemblies, specific overlapping primers (Supplementary Table S2) were designed, and one of the CLV-1 isolates detected herein, SK1 (Supplementary Table S1), was fully resequenced by direct Sanger sequencing of the generated amplicons (Genewiz, Leipzig, Germany). For the determination of the exact 5’ and 3’ ends of the trichovirus genome, RACE assays were applied. For 3’-RACE, a two-step RT-PCR procedure was performed using a virus-specific primer and an oligo(d)T primer with an anchor sequence (Supplementary Table S3) based on a previously described protocol [9]. Likewise, 5’-RACE was performed using the primers described in Supplementary Table S3, following a previously described protocol [9]. All sequences of overlapping fragments were assembled using Geneious Prime® 2022.0.1. Nearly complete CLV-1 genome sequences were analyzed using ORF Finder to determine the genomic organization of the virus and the predicted amino acid (aa) sequences of its gene products. MAFFT v.7.490 was used to make multiple alignments for nucleotide and amino acid sequence comparisons. In the next step, phylogenetic reconstructions were made using all of the CLV-1 isolates characterized in this study, the Georgian CLV-1 isolate, and other related trichovirus sequences available in the GenBank database. The best evolutionary model for each dataset (full genome and protein-encoding genes) was chosen using ModelTest, implemented in IQ-TREE, and phylogenetic relationships were determined by the maximum-likelihood (ML) method in MEGA 7 [10]. The reliability of the phylogenetic hypothesis was evaluated using nonparametric bootstrap analysis (NPB). Finally, 151 sweet cherry leaf samples were collected from three prefectures in northern Greece or obtained from the collection of the Laboratory of Plant Pathology (Aristotle University of Thessaloniki) and tested for the presence of CLV-1. All samples were subjected to total RNA extraction using ‘method A’ as described by Chatzinasiou et al. [11]. For the detection of CLV-1, a new primer set, CLV-1-det-F/CLV-1-det-R (Supplementary Table S2), was designed based on the sequences of the isolates determined by HTS and of the ‘Mskhvil Nakota’ isolate from Georgia (MK770441), amplifying a 438-bp fragment of the CP gene. RT-PCR assays were performed as follows: 10 to 50 ng of total RNA extract was used as template in a reaction containing 10 mM Tris-HCl (pH 8.9), 50 mM KCl, 2.5 mM MgCl_2_, 0.2 mM each dNTP, 0.2 µM each primer, 3 U of MMLV (Invitrogen), 1.5 U of GRS Hot-StartTaq DNA polymerase (GRiSP Research Solutions), and DEPC-treated water to a final volume of 25 µl. The cycling conditions were 45°C for 30 min and 94°C for 5 min, followed by 40 cycles of 94°C for 30 s, 60°C for 30 s, and 72°C for 20 s, and then a final extension at 72°C for 2 min. Amplicons obtained from 15 selected isolates (including the ones obtained through HTS) from different collection years/locations (Supplementary Table S4) were sequenced directly from both ends (Genewiz), and sequences of overlapping fragments were assembled using Geneious Prime® 2022.0.1. All 151 samples were tested further using the primer set P3CLV1DetF1/P3CLV1DetR1 [8], which is specific for the Georgian isolate of CLV-1, according to the protocols and conditions described by the authors, in order to investigate the inclusiveness of this primer pair and whether isolates similar to the Georgian one might be present in Greek orchards.

*De novo* assembly of reads from seven out of eight HTS sequenced sweet cherry samples produced contigs, ranging from 216 to 4,297 nt in length, that shared 82.46–83.82% nucleotide (nt) sequence identity with the reference CLV-1 isolate (Supplementary Table S1). No evidence for viral infection was found in sample CAV4, but all of the other samples analyzed were also infected by little cherry virus 1 and, depending on the cherry sample, of an additional zero (sample 170) to four (sample C18) already known viruses, such as prunus virus I, cherry virus A and F, prunus virus F, and cherry necrotic rusty mottle virus (Supplementary Table S1). Iterative mapping of reads to the assembled contigs allowed nearly complete genome sequences to be obtained for all of the CLV-1 isolates. Isolate SK1 (OP750483) was selected for Sanger resequencing in order to confirm the quality of the *de novo* assembly, and 5’ and 3’ RACE assays were successfully implemented to determine its extreme 5′ and 3′ genome ends. The assembled, nearly complete genome sequences of the seven Greek CLV-1 isolates ranged from 7,458 to 7,476 nt in length (accession numbers OP750481-7), excluding the poly-A tail, indicating that very little sequence information was missing from the *de novo*-assembled genome sequences when compared to the fully resequenced SK1 genome. The complete SK1 genome is 7,458 nt in length and contains three overlapping open reading frames (ORF) organized in the same arrangement as those of CLV-1 [8]. Briefly, ORF1 (5,619 nt) codes for the putative replicase (1872 aa, 215 kDa), which includes methyltransferase (aa 43–336), 2OG-Fe(II) oxygenase (aa 723–826), peptidase-C34 (aa 831–922), helicase (aa 1046–1294), and RNA-dependent RNA polymerase (aa 1460–1774) domains. ORF2 (1,371 nt) and ORF3 (579 nt) encode a putative movement protein (456 aa, 51 kDa) and a putative capsid protein (CP) (192 aa, 21 kDa), respectively.

*In silico* analysis of the terminal regions of all seven Greek CLV-1 isolates revealed slight differences in the 5’ untranslated region (5’-UTR) of the isolates 170, K6, K7, K18, and C18 compared to the isolates SK1 and C118. More specifically, isolates SK1 and C118 contain a 12-nt long sequence that is identical to that found in the ‘Mskhvil Nakota’ CLV-1 isolate and in other trichoviruses, such as ACLSV, CMLV, and peach mosaic virus (PcMV) (Fig. 1A). On the other hand, isolates 170, K6, K7, K18, and C18 have a 15-nt long sequence in common in their 5’-UTR that is identical to that seen in members of unrelated plant virus genera, such as potyviruses, cytorhabdoviruses, ilarviruses, and cileviruses (Fig. 1B). Two CLV-1 isolates, K6 and SK1, were subjected to 5’RACE assays, and subsequent Sanger sequencing verified the presence of these divergent nt sequences. The UTRs of plant viruses are known to be involved in efficient virus replication and translation, and whether these similarities and/or differences in the 5’-UTR of CLV-1 might have an effect on the outcome of these processes remains to be investigated.

**Fig. 1:**
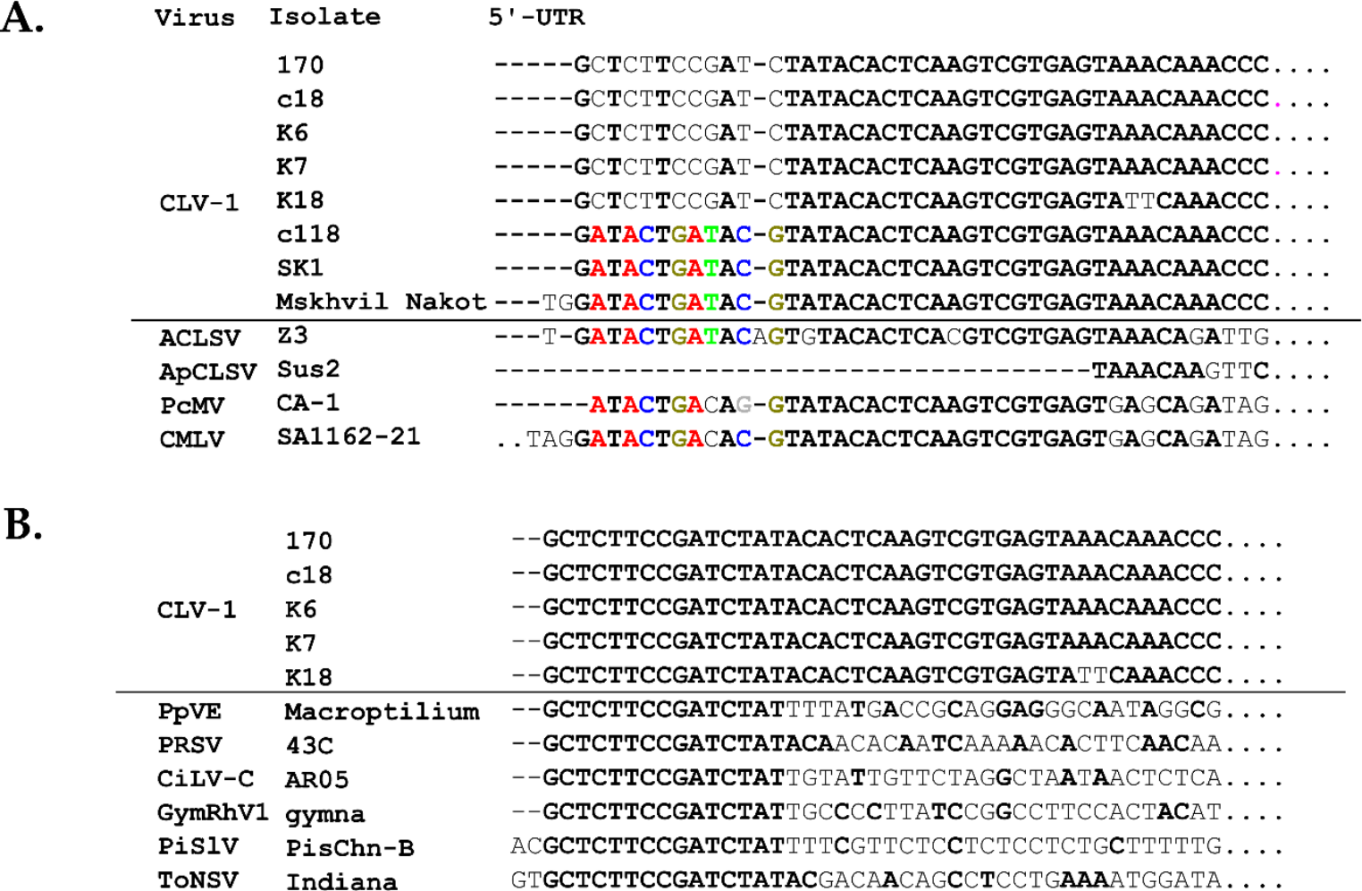
Multiple alignment of the 5’-terminal nucleotide sequences of cherry latent virus 1 (CLV-1) isolates and other related or unrelated viruses. (**A**) Conserved nucleotides (shown in color) detected in the 5’UTR of three CLV-1 isolates (C118, SK1, ‘Mskhvil Nakot’) and related trichoviruses. (**B**) A conserved 15-nt sequence (shown in black, bold, capital letters) located in the 5’UTR of five CLV-1 isolates (170, C18, K6, K7, K18) and unrelated plant viruses. Mismatches to CLV-1 isolates are indicated by grey letters. ACLSV, apple chlorotic leaf spot virus (JN634761); APCLSV, apple pseudo-chlorotic leaf spot virus (NC_006946); PcMV, peach mosaic virus (NC_011552); CMLV, cherry mottle leaf virus (NC_002500); PpVE, papaya virus E (MW239071); PRSV, papaya ringspot virus (KX655869); CiLV-C, citrus leprosis virus C (MT554541, RNA2); GymRhV1, Gymnadenia rhellicani virus 1 (MW328732); PiSlV, pistacia sobemo-like virus (MT334603); ToNSV, tomato necrotic spot virus (MH780155, RNA2)

Comparisons of the genome sequences of the Greek CLV-1 isolates revealed that they were 94.8–98.6% identical to each other and only 81.7–82.4% identical to that of the Georgian ‘Mskhvil Nakota’ isolate (Supplementary Table S5). Similar percentages were observed when individual ORFs and their putatively encoded proteins were compared (Supplementary Tables S6-S8). Subsequent phylogenetic analysis based on the complete genome sequences of CLV-1 isolates and those of related trichoviruses, as well as phylogenetic reconstructions based on the amino acid sequences of their various proteins, demonstrated that the Greek isolates form a separate, coherent, and bootstrap-supported clade within CLV-1 (Fig. 2). Taken together, it may be suggested that they represent a new divergent group of isolates of CLV-1 present in Greek sweet cherry orchards.

**Fig. 2:**
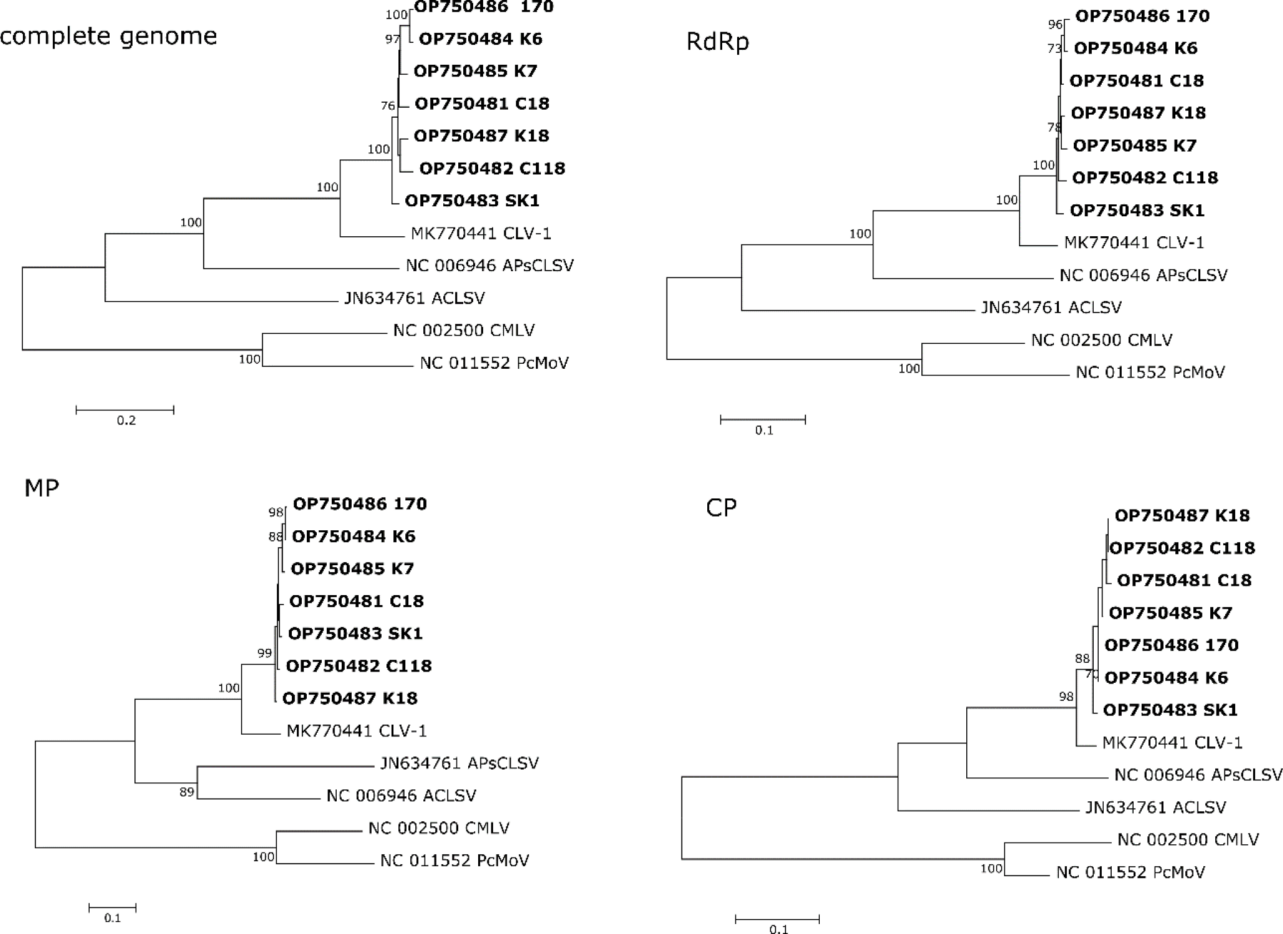
Phylogenetic analysis based on complete genome sequences and amino acid sequences of the RdRp, MP, and CP proteins of cherry latent virus 1 (CLV-1) and other trichoviruses. The best-fit model for each alignment was calculated, and the phylogenetic trees were constructed using the maximum-likelihood method. The statistical significance of branches was evaluated by bootstrap analysis (500 replicates). Bootstrap values above 70% are indicated. ACLSV, apple chlorotic leaf spot virus (JN634761); APCLSV, apple pseudo-chlorotic leaf spot virus (NC_006946); PcMV, peach mosaic virus (NC_011552); CMLV, cherry mottle leaf virus (NC_002500)

Leaf samples from 26 sweet cherry orchards and two nurseries were then tested using a specific RT-PCR assay with the primer set CLV-1-det-F/CLV-1-det-R, which was designed to have broad specificity (Supplementary Table S2). Although sweet cherries are cultivated both on the mainland and on the islands of Greece, 65–70% of the cherry production is located in northern Greece, in the prefectures Imathia and Pella [12]. For this reason, we tested samples from those regions. Molecular assays revealed the presence of CLV-1 in 31.1% (47/151) of the samples tested. In particular, CLV-1 was detected in sweet cherry samples collected from 18 orchards corresponding to three different geographic regions (Imathia, Pella, and Pieria) (47/126), whereas the virus was absent from sweet cherry samples from a nursery (0/25) (Table 1). Amplicon sequencing was performed for eight isolates collected from different orchards and seven isolates analyzed by HTS (Supplementary Table S4). The results revealed that the Greek isolates share pairwise identity levels of 95.2–100% in the amplicon sequence but only 87.7–88.8% with the Georgian ‘Mskhvil Nakota’ isolate. It is noteworthy that the virus was also detected in samples C18, C118, and SK1, which were collected as far back as 2009 and 2014 (Supplementary Table S4). We can therefore safely conclude that the virus has been present in Greek orchards for at least 13 years but its presence remained unnoticed. It should be mentioned that all of the samples collected, with the two exceptions (C18 and C118), originated from cherry trees that did not exhibit any symptoms associated with viral infection, which tends to confirm the initial hypothesis that CLV-1 is probably latent in sweet cherry [8]. Nevertheless, putative differences in the pathogenicity of CLV-1 in various cultivars and its interaction with other viruses remain open questions.

**Table 1:** Incidence of cherry latent virus 1 in sweet cherry in different geographic regions and the years of collection. Plant material that originated from orchards is indicated by “Ch”, whereas that from nurseries is indicated by “ChN”.

Orchard ID	Geographic location	Year of collection	Number of positive samples/ number of total samples	Cherry cultivar
Ch1	Imathia	2009	2/30	Ferrovia
Ch2	Pella	2014	1/1	Larian
Ch3	Pieria	2014	3/5	Bakirtzeika
Ch4	Imathia	2014	1/3	Bakirtzeika
Ch5	Imathia	2014	3/5	Bakirtzeika
Ch6	Pella	2014	1/4	Bakirtzeika
Ch7	Pella	2014	0/4	Bakirtzeika
Ch8	Pella	2014	3/3	Bakirtzeika
Ch9	Pella	2014	0/4	Bakirtzeika
Ch10	Pella	2019	0/4	Tragana Edessis
Ch11	Pella	2019	3/4	Tragana Edessis
Ch12	Pella	2019	2/3	Tragana Edessis
Ch13	Pella	2019	3/3	Tragana Edessis
Ch14	Pella	2019	3/4	Tragana Edessis
Ch15	Pella	2019	2/3	Tragana Edessis
Ch16	Pella	2019	3/3	Tragana Edessis
Ch17	Pella	2019	1/3	Tragana Edessis
Ch18	Pella	2019	0/4	Tsolakeiko
Ch19	Imathia	2020	0/4	Bigarreau Burlat
Ch20	Imathia	2020	0/2	B.S.H. Giant
Ch21	Imathia	2020	2/2	Larian
Ch22	Imathia	2020	0/2	Petrokeraso Achaias
Ch23	Imathia	2020	2/2	Tragana Edessis
Ch24	Imathia	2020	1/2	Van
Ch25	Pella	2020	0/10	Tsolakeiko
Ch26	Pella	2020	7/10	Tsolakeiko
ChN1	Imathia	2019	0/15	Carmen
ChN2	Imathia	2020	0/10	Ferrovia

The published primer set P3CLV1DetF1/P3CLV1DetR1 [8] designed to amplify part of the ORF1 of the Georgian CLV-1 isolate was also evaluated. Preliminary *in silico* analysis, based on an alignment of the sequences of the Georgian and Greek isolates, revealed nine and eight mismatches in the forward and reverse primer, respectively (data not shown). Nonetheless, all 151 survey samples were analyzed using these primers but no amplification was observed, suggesting that isolates related to the Georgian isolate may not be present in Greece.

In summary, this work highlights the identification and molecular characterization of a divergent group of CLV-1 isolates with the aid of HTS technology, thus further advancing our knowledge about the genetic variability of this virus. So far, it remains unclear whether or to what extent CLV-1 may impact sweet cherry production. Nevertheless, its high prevalence in several Greek orchards suggests the need for further systematic inspections and offers novel opportunities to study its epidemiological properties, mechanism of spread, and potential pathogenicity in the near future.

### Electronic Supplementary Material

Below is the link to the electronic supplementary material

Supplementary Material 1
