# Supplementary material for: Identification of divergent isolates of cherry latent virus 1 in Greek sweet cherry orchards
Source: Arch Virol. 2023 Sep 7;168(10):243. doi: 10.1007/s00705-023-05875-7 (PMC10485082; doi:10.1007/s00705-023-05875-7)
Supplement: Supplementary file 1 — Supplementary Material 1 [file 705_2023_5875_MOESM1_ESM.docx]

**Supplementary files**

**Table S1.** Description of analyzed samples and viral species identified with high throughput sequencing (HTS). Geographic origin, year of collection, contig counts and length for CLV-1 using the BLASTn analysis of the data set derived from *de novo* assembly and other viruses detected with HTS are indicated. *Abbreviations*: LChV1: Little cherry virus 1; PrVI: Prunus virus I; CVF: Cherry virus F; PrVF: Prunus virus F; CVA: Cherry virus A; CNRMV: Cherry necrotic rusty mottle virus; na: not applicable.

| **Sample ID** | **Cultivar** | **Orchard ID/Geographic region** | **Year of collection** | **Reads after trimming** | **Reads after host genome removal** | **Number of trichovirus contigs** | **Contig length(s)** | **% nt identity* with CLV-1 (MK770441)** | **Other viruses present** | | | | | |
| --- | --- | --- | --- | --- | --- | --- | --- | --- | --- | --- | --- | --- | --- | --- |
|  |  |  |  |  |  |  |  |  | **LChV1** | **PrVI** | **CVF** | **PrVF** | **CVA** | **CNRMV** |
| 170 | Tragana Edessis | Ch23/Imathia | 2020 | 5,033,697 | 1,466,707 | 6 | 290-1567 | 82.76 | + | - | - | - | - | - |
| C118 | Larian | Ch2/Pella | 2014 | 11,230,661 | 6,456,980 | 8 | 250-1570 | 83.18 | + | - | - | - | + | + |
| C18 | Ferrovia | Ch1/Imathia | 2009 | 14,001,093 | 2,828,680 | 2 | 663-3800 | 83.58 | + | + | + | - | + | + |
| K18 | Tragana Edessis | Ch14/Pella | 2020 | 5,058,816 | 1,057,568 | 19 | 222-1347 | 83.82 | + | - | + | + | + | - |
| K6 | Tragana Edessis | Ch11/Pella | 2019 | 4,900,358 | 1,119,105 | 7 | 284-3358 | 82.46 | + | - | + | + | - | - |
| K7 | Tragana Edessis | Ch11/Pella | 2019 | 4,757,631 | 946,935 | 5 | 216-4285 | 82.57 | + | - | + | + | - | - |
| SK1 | Bakitrzeika | Ch6/Pella | 2014 | 5,544,849 | 1,347,277 | 1 | 4297 | 83.45 | + | - | + | + | - | - |
| CAV4 | Carmen | ChN1/Imathia | 2019 | 4,073,785 | 1,195,964 | 0 | na | na | - | - | - | - | - | - |

*The longest contig corresponding to each virus was used in BLASTn analysis.

**Table S2.** Primers used for Sanger sequencing of CLV-1.

| **Primer name** | **Primer sequence (5’🡪3’)** | **Ta (^o^C)** | **Amplicon length (bp)** |
| --- | --- | --- | --- |
| CLV1-F1 | GATACTGATACGTATACACTCAAG | 54 | 933 |
| CLV1-R1 | GAAAGAGATGATGGGACCC |  |  |
| CLV1-F2 | AGTTGGTGTACATGCCCGA | 55 | 959 |
| CLV1-R2 | ATTCCTCTTTCCACTCAAGG |  |  |
| CLV1-F3 | TTGTGGATGTGATCAAAGGTAC | 55 | 905 |
| CLV1-R3 | TCCTCATTGTCTCTGTGCATC |  |  |
| CLV1-F4 | TGCCTTGATTATCCCATGGTCTA | 55 | 884 |
| CLV1-R4 | TTGAGAGAAACGATACCTGTAG |  |  |
| CLV1-F5 | TCAAGGAGAACGAACTTGGATTC | 57 | 917 |
| CLV1-R5 | TGTTCCCTTGCTTTTCCATGAAG |  |  |
| CLV1-F6 | ATGTGACTGGTTTGAATGATTAC | 55 | 876 |
| CLV1-R6 | TTGATTTTGCCCCTCTGAATTTG |  |  |
| CLV1-F7 | TTACAAGGTCAATCAAGCCATG | 56 | 917 |
| CLV1-R7 | TCACTTTAGCCTTGAGACTCAG |  |  |
| CLV1-F8 | CAAATATGGCTTTCACCTTCTG | 55 | 928 |
| CLV1-R8 | TTATAGAATTTGCTAGATCAGGATC |  |  |
| CLV1-F9 | ATGCTCTATTCAAGAAGAACGC | 56 | 809 |
| CLV1-R9 | GTATCCACTTTGTTCCTCAGGT |  |  |
| CLV1-F10 | ACTCTGTGTCTAGTCTCTCAAGC | 55 | 720 |
| 28V^1^ | CACGGATCCCGGGTTTTTTTTTTTTTTTTTV |  |  |
| CLV-1-det-F | ATCGCAATCCAAGGAACCTCCGA | 60 | 438 |
| CLV-1-det-R | GAGATCGCCCGTAACAGCAGAAAG |  |  |

^1^Gibbs & Mackenzie (1997)

**Table S3.** Primers used for 5’ and 3’ RACE analysis of CLV-1 isolate SK1.

| **Primer use** | **Primer name** | **Primer sequence (5’🡪3’)** | **Ta**  **(^o^C)** |
| --- | --- | --- | --- |
| 5’ first PCR | 5-Tricho-R1 | TGGCGTTCTGTAGGAGAATG | 55 |
|  | AAP^1^ | GGCCACGCGTCGACTAGTACGGGIIGGGIIGGGIIG |  |
| 5’ nested PCR | 5-Tricho-R1n | ATGCCATAGTGGTATCTTAACTTTCG | 59 |
|  | AUAP^1^ | GGCCACGCGTCGACTAGTAC |  |
| 3’ first PCR | 3-Tricho-F1 | GAACCCACGAAAGAGTATAAAG | 55 |
|  | 28V^2^ | CACGGATCCCGGGTTTTTTTTTTTTTTTTTV |  |
| 3’ semi-nested PCR | 3’-Tricho-F1n | AGTCGTGGTTAACAAACGTAACTGG | 61 |
|  | 28V^2^ | CACGGATCCCGGGTTTTTTTTTTTTTTTTTV |  |

^1^ Alves-Freitas et al. (2019), ^2^Gibbs and Mackenzie (1997)

**References**

Alves-Freitas DMT, Pinheiro-Lima B, Faria JC, Lacorte C, Ribeiro SG, Melo FL (2019) Double-stranded RNA high-throughput sequencing reveals a new cytorhabdovirus in a Bean golden mosaic virus-resistant common bean transgenic line. Viruses 11:90.

Gibbs A, Mackenzie A (1997) A primer pair for amplifying part of the genome of all potyvirids by RT-PCR. J Virol Methods 63:9–16.

**Table S4.** CLV1 Isolates used for partial CP gene Sanger sequencing

| **ID code** | **Cultivar** | **Orchard ID / Geographic region** | **Year of collection** | **Accession number** |
| --- | --- | --- | --- | --- |
| C18 | Ferrovia | Ch1/Imathia | 2009 | OP750481 |
| C118 | Larian | Ch2/Pella | 2014 | OP750482 |
| SK1 | Bakitrzeika | Ch6/Pella | 2014 | OP750483 |
| K6 | Tragana Edessis | Ch11/Pella | 2019 | OP750484 |
| K7 | Tragana Edessis | Ch11/Pella | 2019 | OP750485 |
| 170 | Tragana Edessis | Ch23/Imathia | 2020 | OP750486 |
| K18 | Tragana Edessis | Ch14/Pella | 2020 | OP750487 |
| TsAR1 | Tsolakeiko | Ch26/Pella | 2020 | OP750488 |
| RA1 | Bakirtzeika | Ch3/Pieria | 2014 | OP750489 |
| GI3 | Bakirtzeika | Ch4/Imathia | 2014 | OP750490 |
| SU1 | Bakirtzeika | Ch8/Pella | 2014 | OP750491 |
| K3 | Tragada Edessis | Ch10/Pella | 2019 | OP750492 |
| K22 | Tragana Edessis | Ch16/Pella | 2019 | OP750493 |
| D133 | Larian | Ch21/Imathia | 2020 | OP750494 |
| D135 | Tragana Edessis | Ch23/Imathia | 2020 | OP750495 |

**Table S5.** Percentages of pairwise complete genome nucleotide identity between CLV-1 isolates or with two related trichoviruses, apricot pseudo-chlorotic leaf spot virus (APCLSV, NC_006946) and apple chlorotic leaf spot virus (ACLSV, JN634761).

|  | 170 | C18 | C118 | K6 | K7 | K18 | SK1 | MK770441 | APCLSV | ACLSV |
| --- | --- | --- | --- | --- | --- | --- | --- | --- | --- | --- |
| 170 |  |  |  |  |  |  |  |  |  |  |
| C18 | 95.4 |  |  |  |  |  |  |  |  |  |
| C118 | 94.8 | 95.4 |  |  |  |  |  |  |  |  |
| K6 | 98.6 | 95.6 | 94.9 |  |  |  |  |  |  |  |
| K7 | 96.2 | 96.4 | 95.4 | 96.3 |  |  |  |  |  |  |
| K18 | 95.6 | 96.6 | 96.1 | 95.7 | 96.4 |  |  |  |  |  |
| SK1 | 95.1 | 95.3 | 95.2 | 95.2 | 95.6 | 95.5 |  |  |  |  |
| MK770441 | 81.8 | 82.1 | 82.1 | 81.7 | 82.1 | 82.0 | 82.4 |  |  |  |
| APCLSV | 66.5 | 67.2 | 66.9 | 66.6 | 67.0 | 66.8 | 66.9 | 66.9 |  |  |
| ACLSV | 63.0 | 63.4 | 63.3 | 63.0 | 63.1 | 63.2 | 63.1 | 62.9 | 63.3 |  |

**Table S6.** Percentages of pairwise ORF1 nucleotide (down-left) and amino acid (top-right) identity between CLV-1 isolates or with two related trichoviruses, apricot pseudo-chlorotic leaf spot virus (APCLSV, NC_006946) and apple chlorotic leaf spot virus (ACLSV, JN634761).

|  | 170 | C18 | C118 | K6 | K7 | K18 | SK1 | MK770441 | APCLSV | ACLSV |
| --- | --- | --- | --- | --- | --- | --- | --- | --- | --- | --- |
| 170 |  | 98.5 | 97.9 | 99.0 | 98.3 | 98.5 | 98.1 | 90.5 | 72.1 | 65.9 |
| C18 | 95.1 |  | 98.5 | 98.8 | 98.7 | 99.0 | 98.2 | 90.7 | 72.3 | 66.0 |
| C118 | 94.5 | 95.1 |  | 98.1 | 98.3 | 98.6 | 98.1 | 90.6 | 72.1 | 65.8 |
| K6 | 98.6 | 95.3 | 94.7 |  | 98.5 | 98.6 | 98.0 | 90.5 | 72.2 | 65.8 |
| K7 | 96.0 | 96.1 | 95.1 | 96.1 |  | 98.8 | 98.1 | 90.8 | 72.5 | 66.1 |
| K18 | 95.4 | 96.4 | 95.89 | 95.5 | 96.2 |  | 98.4 | 90.8 | 72.4 | 66.1 |
| SK1 | 94.7 | 94.8 | 94.7 | 94.9 | 95.2 | 95.2 |  | 91.0 | 72.3 | 66.2 |
| MK770441 | 81.0 | 81.5 | 81.2 | 81.0 | 81.3 | 81.3 | 81.6 |  | 72.2 | 65.7 |
| APCLSV | 67.5 | 68.3 | 68.0 | 67.7 | 68.1 | 68.0 | 68.1 | 68.0 |  | 65.3 |
| ACLSV | 64.4 | 64.8 | 64.7 | 64.3 | 64.4 | 64.6 | 64.5 | 64.0 | 63.8 |  |

**Table S7.** Percentages of pairwise ORF2 nucleotide (down-left) and amino acid (top-right) identity between CLV-1 isolates or with two related trichoviruses, apricot pseudo-chlorotic leaf spot virus (APCLSV, NC_006946) and apple chlorotic leaf spot virus (ACLSV, JN634761).

|  | 170 | C18 | C118 | K6 | K7 | K18 | SK1 | MK770441 | APCLSV | ACLSV |
| --- | --- | --- | --- | --- | --- | --- | --- | --- | --- | --- |
| 170 |  | 97.1 | 97.6 | 99.6 | 98.5 | 97.1 | 97.8 | 84.4 | 55.5 | 54.0 |
| C18 | 96.1 |  | 98.2 | 97.6 | 97.8 | 97.8 | 98.7 | 84.0 | 55.7 | 53.7 |
| C118 | 96.0 | 96.4 |  | 98.0 | 98.2 | 98.7 | 98.7 | 85.3 | 56.4 | 53.7 |
| K6 | 98.2 | 96.3 | 95.8 |  | 98.9 | 97.6 | 98.2 | 84.6 | 55.7 | 53.7 |
| K7 | 96.7 | 97.0 | 96.6 | 96.6 |  | 97.8 | 98.5 | 84.6 | 55.3 | 53.7 |
| K18 | 95.8 | 96.8 | 97.3 | 95.9 | 97.0 |  | 98.2 | 85.1 | 56.2 | 54.0 |
| SK1 | 96.5 | 96.4 | 96.2 | 96.4 | 96.7 | 96.4 |  | 84.9 | 55.7 | 53.7 |
| MK770441 | 83.3 | 83.3 | 84.0 | 82.9 | 83.8 | 83.4 | 83.7 |  | 56.8 | 54.8 |
| APCLSV | 65.1 | 65.3 | 64.9 | 65.0 | 65.1 | 65.0 | 64.7 | 64.7 |  | 59.1 |
| ACLSV | 63.1 | 62.7 | 62.9 | 63.1 | 63.0 | 62.5 | 62.3 | 62.5 | 65.9 |  |

**Table S8.** Percentages of pairwise ORF3 nucleotide (down-left) and amino acid (top-right) identity between CLV-1 isolates or with two related trichoviruses, apricot pseudo-chlorotic leaf spot virus (APCLSV, NC_006946) and apple chlorotic leaf spot virus (ACLSV, JN634761).

|  | 170 | C18 | C118 | K6 | K7 | K18 | SK1 | MK770441 | APCLSV | ACLSV |
| --- | --- | --- | --- | --- | --- | --- | --- | --- | --- | --- |
| 170 |  | 98.4 | 99.0 | 100 | 99.5 | 99.0 | 99.0 | 95.3 | 74.6 | 69.9 |
| C18 | 97.9 |  | 99.5 | 98.4 | 99.0 | 99.5 | 97.9 | 94.8 | 75.6 | 70.5 |
| C118 | 97.4 | 98.8 |  | 99.0 | 99.5 | 100 | 98.4 | 95.3 | 75.1 | 70.5 |
| K6 | 100 | 97.9 | 97.4 |  | 99.5 | 99.0 | 99.0 | 95.3 | 74.6 | 69.9 |
| K7 | 98.4 | 98.4 | 97.9 | 98.4 |  | 99.5 | 98.4 | 94.8 | 74.6 | 69.9 |
| K18 | 97.8 | 99.1 | 99.0 | 97.8 | 98.3 |  | 98.4 | 95.3 | 75.1 | 70.5 |
| SK1 | 98.1 | 98.8 | 98.3 | 98.1 | 98.3 | 98.6 |  | 95.8 | 74.6 | 70.5 |
| MK770441 | 89.5 | 88.9 | 89.1 | 89.5 | 89.1 | 89.1 | 89.1 |  | 74.6 | 71.0 |
| APCLSV | 71.8 | 72.5 | 72.0 | 71.8 | 71.3 | 72.2 | 71.8 | 73.7 |  | 68.4 |
| ACLSV | 68.7 | 69.8 | 69.8 | 68.7 | 68.7 | 69.9 | 69.4 | 69.8 | 70.3 |  |
